# Carrier-Based Application of Phyto-Benefic and Salt-Tolerant *Bacillus wiedmannii* and *Bacillus paramobilis* for Sustainable Wheat Production Under Salinity Stress

**DOI:** 10.3390/plants14142096

**Published:** 2025-07-08

**Authors:** Raina Rashid, Atia Iqbal, Muhammad Shahzad, Sidra Noureen, Hafiz Abdul Muqeet

**Affiliations:** 1 Department of Microbiology and Molecular Genetics, The Women University, Multan 66000, Pakistan; rainarashid14@gmail.com (R.R.); sidranoureen911@gmail.com (S.N.); 2 Department of Electrical Engineering, Muhammad Nawaz Sharif University of Engineering and Technology, Multan 66000, Pakistan; 3 Department of Engineering and Technology, Punjab Tianjin University of Technology, Lahore 53720, Pakistan

**Keywords:** *Bacillus* sp., peat, compost, bio-fertilizer, salt-tolerant PGPR, *Triticum aestivum*, *Bacillus wiedmannii*, *Bacillus paramobilis*

## Abstract

Plant growth-promoting rhizobacteria (PGPR) are beneficial soil microorganisms that enhance plant growth and stress tolerance through various mechanisms, including phytohormone production, EPS production, phosphate solubilization, and extracellular enzyme production. These bacteria establish endosymbiotic relationships with plants, improving nutrient availability and overall crop productivity. Despite extensive research on PGPR isolation, their practical application in agricultural fields has faced challenges due to environmental stresses and limited survival during storage. To address these limitations, the present study aimed to isolate salt-tolerant bacterial strains and formulate them with organic carriers to enhance their stability and effectiveness under saline conditions. The isolated bacterial strains exhibited high salt tolerance, surviving NaCl concentrations of up to 850 millimolar. These strains demonstrated basic key plant growth-promoting traits, including phosphate solubilization, auxin production, and nitrogen fixation. The application of carrier-based formulations with both strains, *Bacillus wiedmannii* (RR2) and *Bacillus paramobilis* (RR3), improved physiological and biochemical parameters in wheat plants subjected to salinity stress. The treated plants, when subjected to salinity stress, showed notable increases in chlorophyll a (73.3% by Peat + RR3), chlorophyll b (41.1% by Compost + RR3), carotenoids (51.1% by Peat + RR3), relative water content (77.7% by Compost + RR2), proline (75.8% by compost + RR3), and total sugar content (12.4% by peat + RR2), as compared to the stressed control. Plant yield parameters such as stem length (35.1% by Peat + RR3), spike length (22.5% by Peat + RR2), number of spikes (67.6% by Peat + RR3), and grain weight (39.8% by Peat + RR3) were also enhanced and compared to the stressed control. These results demonstrate the potential of the selected salt-tolerant PGPR strains (ST-strains) to mitigate salinity stress and improve wheat yield under natural field conditions. The study highlights the significance of carrier-based PGPR applications as an effective and sustainable approach for enhancing crop productivity in saline-affected soils.

## 1. Introduction

As recent studies show, more than 20% of the world’s soils are affected by salt due to human activities and climate variability, resulting in a 50% decrease in crop production. With the world’s population growing rapidly, we will need to increase food production by 70% by 2050 [1]. Wheat is an essential crop in terms of providing calories, carbohydrates, and essential nutrients that are important for our health, and it can feed around 36% of the world’s population [2]. Wheat production can contribute approximately 1.7% to the country’s GDP, accounting for about 9.1% of the total agricultural production. Notably, Pakistan’s annual wheat production reaches 27 million tons. This substantial output showcases the crucial role of wheat in meeting both domestic consumption and potential export demands [3].

One of the main reasons behind the low crop yield in Pakistan is the use of organic fertilizers and the presence of salinity in the soil [4]. Salinity stress damages proteins, cell walls, and the nucleus of plant cells by excessive production of reactive oxygen species (ROS) that ultimately lowers the yield and will disturb the Na^+^/K^+^ ratio, causing osmotic imbalance in plants. Along with this, the presence of Na^+^ and Cl^−^ ions can cause a deficiency of nutrients and ion toxicity that ultimately affect the physiological conditions of plants [5]. One specific area of focus is the use of plant growth-promoting rhizobacteria (PGPR) to enhance plant growth under salt stress, which is considered an emerging technology that plays a crucial role in inducing salt stress resistance through multifaceted mechanisms. Previous studies have highlighted the positive impact of Gram-positive *Bacillus* sp. on plant growth under stress by enhancing the production of plant hormones and salt-resistant genes [6]. The real milestone is the gap between the laboratories and the farmers that can be reduced by the application of the PGPR in a portable way that can easily be used in the fields. For this purpose, using organic carriers for bacterial field application is the most affordable, environmentally friendly, and effective approach.

Recent studies have identified *Bacillus wiedmannii* and *Bacillus paramobilis* as highly effective bacterial strains that significantly enhance plant physiological and biochemical parameters under salt stress conditions. These bacterial species exhibit multiple plant-beneficial traits, including phosphate solubilization, nitrogen fixation, production of stress-alleviating phytohormones, and secretion of antioxidant enzymes [7].

*Bacillus wiedmannii* has been reported to stimulate root elongation, enhance nutrient uptake, and improve photosynthetic efficiency by modulating stress-responsive gene expression [8]. Additionally, *Bacillus paramobilis* contributes to salt stress tolerance by improving osmotic balance, increasing proline accumulation, and reducing ROS-induced oxidative damage. In wheat, applying *Bacillus wiedmannii* and *Bacillus paramobilis* has been shown to significantly increase chlorophyll content, relative water content (RWC), and sugar accumulation under saline conditions. These bacteria help maintain cell membrane integrity by reducing electrolyte leakage and promoting the synthesis of compatible solutes, which enhance water retention and sustain metabolic activities during stress [9]. In addition to their role in stress mitigation, *Bacillus wiedmannii* and *Bacillus paramobilis* also possess biocontrol properties against soil-borne pathogens. Their ability to produce antimicrobial compounds and induce systemic resistance in plants make PGPR a valuable candidate for sustainable agricultural practices [10]. These findings highlight the dual benefits of ST-PGPR, which not only enhance stress tolerance but also contribute to disease suppression, offering a holistic approach to crop improvement.

Salt-tolerant plant growth-promoting rhizobacteria (ST-PGPR) mitigate salt stress in plants through both direct and indirect regulatory mechanisms. Direct regulation involves the synthesis of phytohormone signals, such as auxin, gibberellins, ethylene, and abscisic acid production [11], chemical processes like nitrogen fixation, and enhanced nutrient uptake. Indirect regulation is achieved by bolstering the plant’s defense system and modulating metabolic pathways that facilitate the accumulation of osmoregulatory and antioxidant substances. These combined mechanisms contribute to the alleviation of salt stress and promote plant growth and resilience [12]. Another important trait possessed by *Bacillus* sp. is the ability to form biofilms. Biofilm formation serves as a coating around bacteria that enables them to survive under harsh conditions and also helps in the colonization of plants. Recent research has shown that Bacillus sp. forms a lot of biofilm under stress conditions [13].

The direct application of PGPR in the field environment presents several challenges due to harsh and unstable environmental conditions, short shelf life of bacterial inoculants, inadequate processing methods by the farmers, and poor field conditions. To address these issues, a suitable carrier medium is required to assist the application of these PGPR under natural environmental conditions. Many studies use of synthetic carriers like nanoparticles, alginate beads, use of polymer gel, but these techniques are often expensive, labor-intensive, time-consuming, and less practical for large-scale use. In contrast to a synthetic carrier material, the use of an organic carrier is a more adequate, cost-effective, and environmentally friendly approach [14]. The use of organic carriers, i.e., compost and peat, is quite helpful to assist plants with abiotic stresses. This improves soil water-holding capacity, fertility, and nutrient quality and also combats soil salinity. The organic carriers serves as a medium for the inoculation of PGPR and is considered a very cost-effective and eco-friendly fertilizer for plants [15].

The focus of this study is to investigate the potential of *Bacillus wiedmannii* and *Bacillus paramobilis* to enhance wheat growth under salt stress conditions. By integrating these beneficial microbes with organic carrier-based formulations, this research seeks to develop a biologically driven and sustainable strategy for mitigating salinity stress in wheat. Understanding the underlying mechanisms of PGPR-mediated stress tolerance will contribute to the advancement of environment-friendly approaches for increasing wheat yield and ensuring food security in salt-affected regions.

## 2. Results

### 2.1. Isolation and Purification of Bacteria

A total of 50 bacterial strains were isolated from 10 rhizospheric soil samples, out of which 27 were halotolerant (Table 1). Five strains showed the best growth at 850 mM salt concentration and were selected for further characterization [16].

### 2.2. PGP Characterization of Salt-Tolerant Rhizobacteria

To access the plant growth-promoting potential of isolated salt-tolerant rhizobacterial strains, several experiments were conducted to select the strains to be used as a biofertilizer. Initial tests include auxin production, phosphate solubilization, nitrogen fixation, EPS production, HCN production, and ammonia production [17].

**Figure 1:**
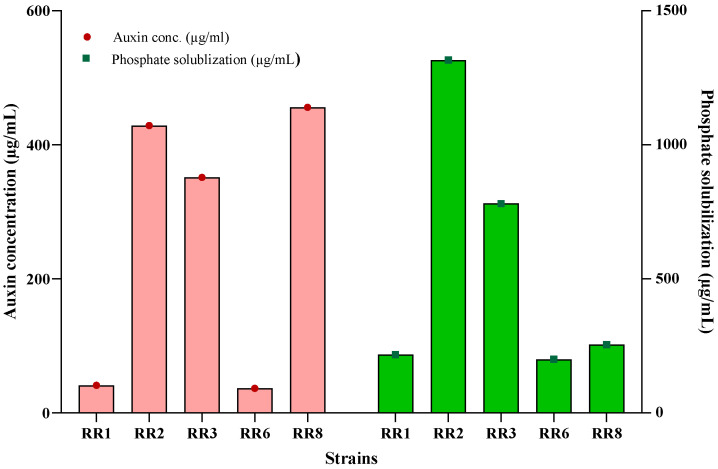
Auxin production and phosphate solubilization by ST-PGPR. Bars represent standard deviation.

All five strains showed positive results for auxin production and phosphate solubilization (Table 2). Auxin production ranged from 36.5 µg/mL (RR6) to 455.7 µg/mL (RR8). The lowest results were shown by RR1 (41.0 µg/mL) and RR6 (36.5 µg/mL), while the other three, RR2 (428.7 µg/mL), RR3 (351.1 µg/mL), and RR6 (455.7 µg/mL), showed maximum production with a sharp contrast to the others (Figure 1).

Phosphate solubilization ranged from 200.1 µg/mL (RR6) to 1316.1 µg/mL (RR2). RR1, RR6, and RR8 showed lower phosphate solubilization indexes (PSIs), that is, 217.2 µg/mL, 200.1 µg/mL, and 255.9 µg/mL, respectively, while RR2 and RR3 showed contrastingly higher PSIs, 1316.1 µg/mL and 780.6 µg/mL, respectively (Figure 1).

All five strains showed positive zones for nitrogen fixation, ranging from 0.86 cm to 2.43 cm (Table 2). The larger zones were shown by RR2 (1.31 cm), RR3 (2.13 cm), and RR8 (2.43 cm), while RR1 (1.13 cm) showed a smaller zone, and the smallest zone was produced by RR6 (0.86 cm). Moreover, 80% of ST-strains were positive for HCN production, 40% showed positive results for ammonia production, and 60% showed positive results for EPS production (Table 2).

Salt-tolerant PGPRs were also evaluated in terms of their extracellular enzymes, such as chitinase, pectinase, catalase, protease, and amylase-producing abilities (Table 3). Concerning chitinase and pectinase, about 80% and 20% of strains showed production, respectively, while none of the strains produced the catalase enzyme. About 60% of strains show reproducibility for protease and amylase enzymes.

The two most potent strains (RR2) and (RR3) regarding plant growth-promoting attributes were selected for further application on wheat to assess the efficiency of their plant–microbe interaction potential when combined with the selected carriers. Moreover, the GC-MS analysis elaborates on the presence of indole compounds and several secondary metabolites in salt-tolerant PGPR (Table 4).

### 2.3. Molecular Characterization of Bacterial Strains

**Figure 2:**
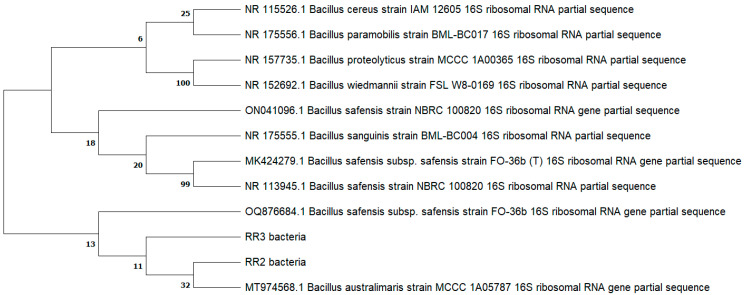
Phylogenetic tree of salt-tolerant strains RR2 and RR3 and neighboring plant growth-promoting bacteria.

The most effective strains, having plant growth-promoting attributes and being salt tolerant, were RR2 and RR3. These strains appeared as damp, opaque, mucoid colonies, were rod-shaped and endospore forming, and were positive for citrate and catalase production. After biochemical analysis, these strains were subjected to 16S rRNA-based identification. RR2 and RR3 were identified as *Bacillus wiedmannii* and *Bacillus paramobilis* with 98% similarity, and their GeneBank accession numbers were PQ057000 and PQ057001, respectively. The phylogenetic tree was constructed using the nucleotide sequence according to the nearest neighboring strains to show an evolutionary relationship (Figure 2).

### 2.4. Characterization of the Carrier and Survival Percentage of PGPR in Carriers

The physiochemical analysis of compost and peat showed electrical conductivities 2.15 and 1.93 dS^−1^, pH 5.6 and 5.28, nitrogen 1.14% and 0.92%, phosphorus 0.97% and 0.64%, potassium 87.61% and 73.56%, moisture 3.89% and 3.37%, and water-holding capacity 28.4% and 31.12%, respectively (Table 5).

The stability of the strains in both carriers was evaluated by storing at room temperature, ranging from 25 °C to 30 °C, for 180 days (6 months). The CFU of RR2 and RR3 decreased gradually over 90 days, from 35.1 × 10^7^ and 30.4 × 10^7^ to 21.0 × 10^7^ and 18.2 × 10^7^, respectively, in compost and from 31.8 × 10^7^ and 29.2 × 10^7^ to 15.9 × 10^7^ and 14.3 × 10^7^, respectively, in peat. However, a sudden decline in the CFU of RR2 and RR3 was noticed during the time interval of 150 days to 180 days, i.e., 7.9 × 10^7^ and 8.3 × 10^7^ in compost and 7.4 × 10^7^ and 5.2 × 10^7^ in peat (Table 6).

### 2.5. Soil Analysis

Soil analysis showed the texture of the soil as sandy loam, with electrical conductivity 2.37 dS^−1^, pH 8.6, available nitrogen, phosphorus, and potassium 91, 4.42, and 98 mg/kg, organic matter 0.25%, and saturation 11% (Table 7).

### 2.6. Seedling Characteristics of Wheat Under Laboratory Conditions

**Figure 3:**
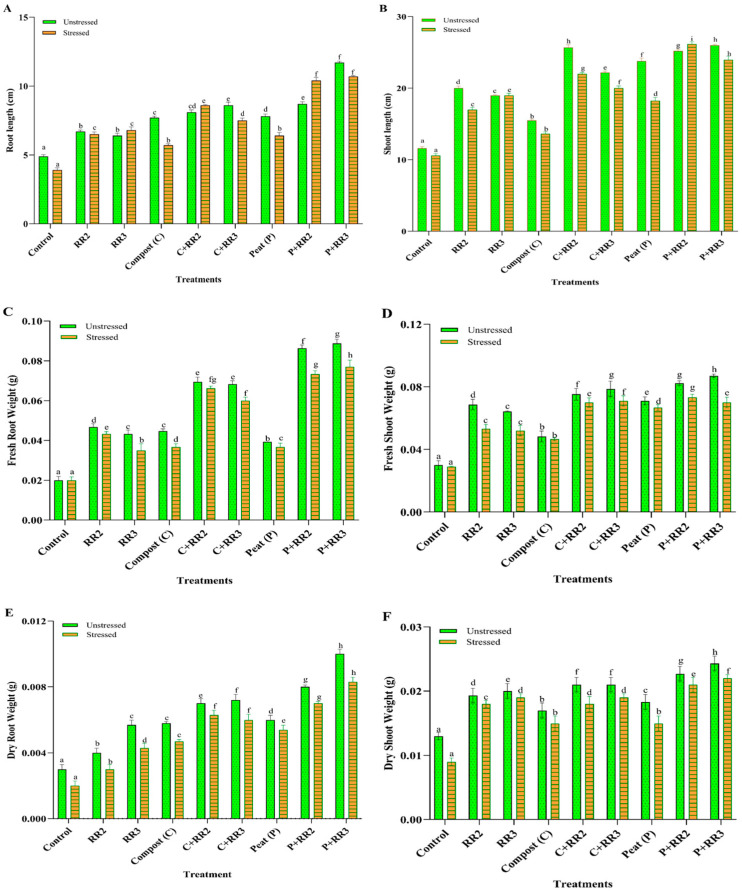
Vegetative parameters of wheat under laboratory conditions. (**A**) Root length, (**B**) Shoot length, (**C**) Fresh root weight, (**D**) Fresh shoot weight, (**E**) Dry root weight, (**F**) Dry shoot weight in stressed and unstressed conditions. All data represented are the means of triplicate values. Bars represent the standard deviation, and those that do not share a letter are significantly different (*p* ≤ 0.05, Tukey’s test).

Salinity adversely affects wheat growth, resulting in a decrease in productivity and biomass. However, the application of salt-tolerant strains, both with and without carriers, significantly improved the yield. There was a visible increase in root length, shoot length, fresh root weight, fresh shoot weight, dry root weight, and dry shoot weight when bacteria along the carrier were applied, as shown in Figure 3. Under unstressed conditions among the tested combinations, the application of peat + *Bacillus paramobilis* (RR3) and peat + *Bacillus weidmannii* (RR2) increased the root length by 58.1% and 48.3%, respectively. However, the other treatments—C + RR2, C + RR3, compost, peat, RR2, and RR3—increased root length by 39.5%, 43.0%, 36.3%, 37.1%, 26.8%, and 23.4%, respectively, as compared to the control. The same trend was observed for shoot length. Maximum shoot length was obtained with the application of peat + RR3 (55.3%) and compost + RR2 (54.8%), respectively, as compared to the control. Other treatments, such as P + RR2, peat, C + RR3, compost, RR2, and RR3, increased the shoot length by 53.9%, 51.2%, 47.7%, 25.1, 42%, and 38.9%, respectively, as compared to the control (Figure 3A). Under stressed conditions, all treatments performed better than under unstressed conditions. The maximum root length was observed by the application of P + RR3 (63.5%) and P + RR2 (62.5%). Other treatments increased root length by 40% (for RR2), 42.6% (for RR3), 31.5% (for compost), 54.6% (for C + RR2), 48% (for C + RR3), and 39% (for peat). The maximum increase in shoot lengths observed by P + RR2 and P + RR3 were 59.4% and 55.7%, respectively. Other treatments increased shoot length—37.6% for RR2, 44.2% for RR3, 22.2% for compost, 51.8% for C + RR2, 47% for C + RR3, and 41.8% for peat—as compared to the stressed control (Figure 3B).

### 2.7. Root Colonization

**Figure 4:**
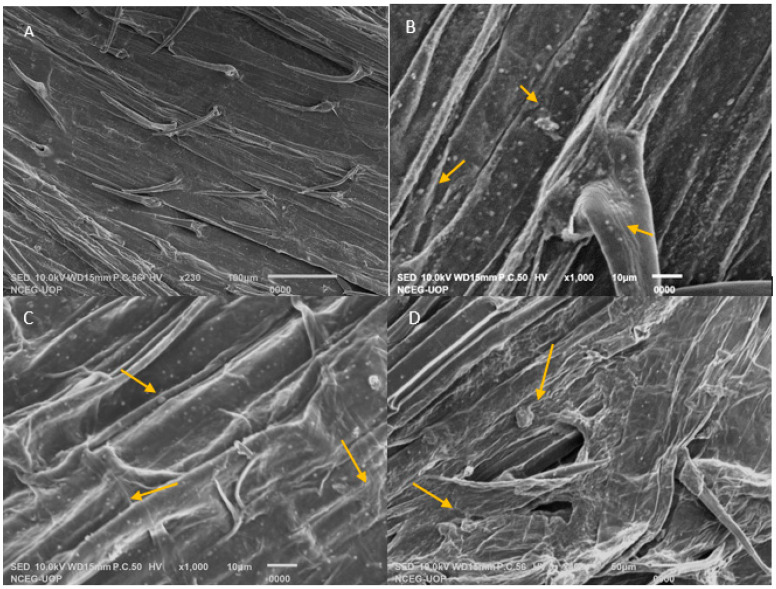
Scanning electron microscopy images of roots of wheat adhering to salt-tolerant PGPRs. (**A**) An uninoculated control has no bacterial colonization; (**B**,**C**) Root colonization RR2 (**D**) Root colonization by RR3. Yellow arrows show the bacterial colonization.

The scanning electron microscopic images show the colonization of RR2 and RR3 on the roots of wheat. There is no colonization present in the control (Figure 4A), while RR2 shows clear colonization (Figure 4B,C) as compared to RR3 (Figure 4D). The selected strains developed very good associations with the roots.

### 2.8. Physiological and Biochemical Parameters of Wheat Under Natural Conditions

**Figure 5:**
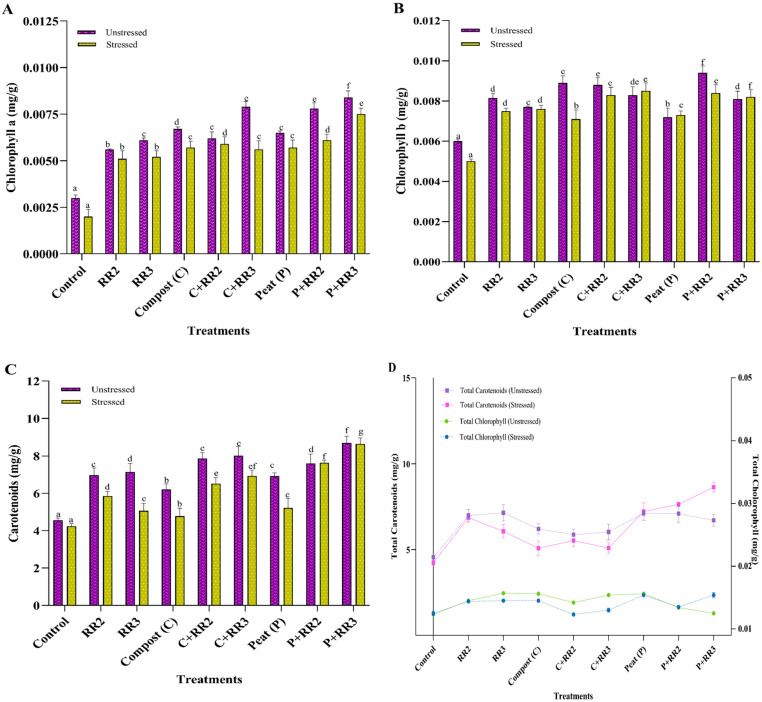
Physiological parameters of wheat under natural conditions. (**A**) Chlorophyll a; (**B**) Chlorophyll b; (**C**) Carotenoids; (**D**) Total chlorophyll and total carotenoids. All data presented in the graph are the means of triplicate values. Bars represent the standard error, and those that do not share a letter are significantly different (*p* ≤ 0.05, Tukey’s test).

Chlorophyll a levels significantly increased in plants treated with bacterial strains *Bacillus wiedmanni* (RR2) and *Bacillus paramobilis* (RR3) and the carriers compost (C) and peat (P). Under stressed conditions, the highest level of chlorophyll a was observed in plants treated with P + RR3, P + RR2, and C + RR2 by 73.3%, 67.2%, and 66.1%, respectively, as compared to control. While under unstressed conditions, maximum chlorophyll a was observed in plants treated with P + RR3 and C + RR3 by 64.2% and 62.0%, respectively, as compared control (Figure 5A). A similar trend was observed for chlorophyll b in wheat leaves; the application of PGPR combined with peat and compost increased the chlorophyll b levels in both stressed and unstressed conditions. Under unstressed conditions, maximum chlorophyll b was observed in P + RR2 by 36.1%, followed by compost, C + RR2, C + RR3, RR2, P + RR3, RR3, and peat by 32.5%, 31.8%, 27.7%, 26.3%, 25.1%, 22.0%, and 16.6%, respectively, as compared to the control. In stressed conditions, the maximum chlorophyll b was observed in C + RR3, P + RR2, P + RR3, and C + RR2 by 41.1%, 40.4%, 39.7%, and 39.0%, respectively, as compared to untreated stressed plants (Figure 5B). The application of both strains in combination with the carriers C + RR2 and P + RR3 had maximum chlorophyll b production as compared to bacterial application alone under stressed condition (Figure 5B). The maximum carotenoids were produced by P + RR3 and C + RR2 by 47.6%, 42.0%, respectively, under unstressed conditions, as compared to control. P + RR3 showed a maximum increase of 51.1% in the presence of salt, as compared to the untreated stressed control. Under stressed conditions, both strains in combination with peat (P + RR2 and P + RR3) gave better carotenoid production than RR2 and RR3 (Figure 5C). A similar trend was observed under unstressed conditions. Peat gave better results compared to individual applications of bacterial strains for carotenoid production (Figure 5D). C + RR2 and C + RR3 performed well under unstressed conditions; however, under stressed conditions, the highest results were observed for P + RR3 for total chlorophyll content (Figure 5D).

**Figure 6:**
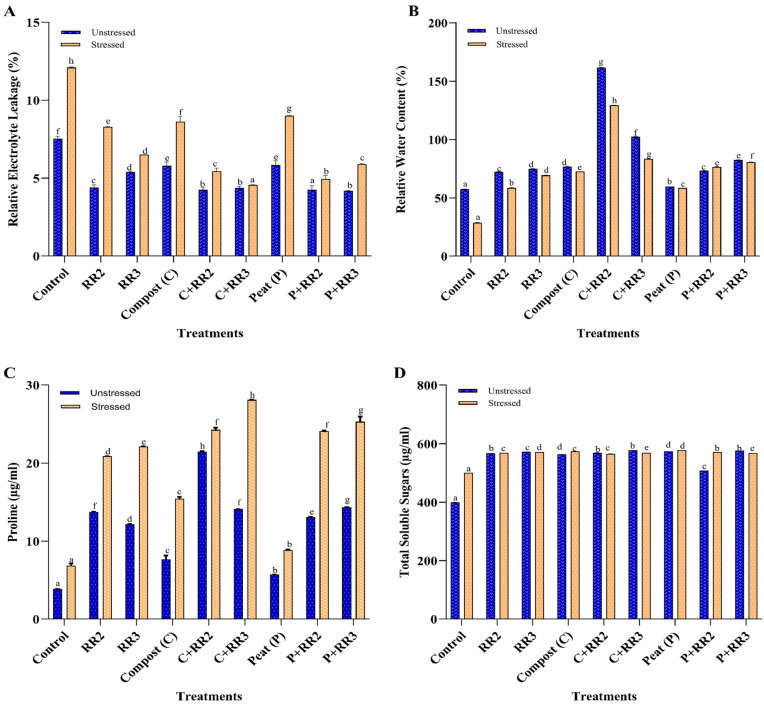
Effect of biochemical parameters on wheat under natural conditions. (**A**) Relative electrolyte leakage; (**B**) Relative water content; (**C**) Proline content; (**D**) Total soluble sugar. All data presented in the graph are means of triplicate values. Bars represent the standard error and those that do not share a letter are significantly different (*p* ≤ 0.05, Tukey’s test).

The REL in the plants increased when subjected to stress for all treatments; however, if a comparison was made between the stressed and unstressed group, less REL was observed in the unstressed group for all treatments. The REL was reduced to 165.5% and 144.8% for C + RR3 and P + RR2 under a saline environment, as compared to untreated salt stress (Figure 6A). The RWC of the stressed plants significantly increased with the application of *Bacillus wiedmannii* (RR2) and *Bacillus paramobilis* (RR3) in combination with compost by 77.7% to 65.5%, respectively, as compared to the control (Figure 6B). The estimated increases for both strains RR2 and RR3 in combination with peat were 62.4% and 64.2%, respectively, compared to the control (Figure 6B).

Proline content and total soluble sugar content in the leaves also increased significantly by the application of strains with carriers. Proline production was increased to 82.5%, 73.9%, and 72.6% for C + RR2, P + RR3, and C + RR3, and 70.62%, 71.9%, and 68.3% for P + RR2, RR2, and RR3, respectively, under unstressed conditions, as compared to the control. However, under salt stress, the maximum proline content was observed in C + RR3, P + RR3, C + RR2, and P + RR2 and was 75.8%, 73.6%, 71.8%, and 71.6% respectively, as compared to untreated stressed plants. Under stressed conditions, RR2 and RR3 in combination with compost gave more proline production as compared to individual applications of RR2 and RR3. Similarly, RR2 and RR3 in combination with peat had better proline production as compared to individual bacterial application (Figure 6C). The total soluble sugar content (TSS) in the leaves showed a constant increase in all treatments as compared to the control. The maximum TSS produced by C + RR3, P + RR3, and peat was 30.8%, 30.6%, and 30.4%, as compared to the unstressed control; however, in the case of stressed plants, the maximum TSS observed by the application of peat, C + RR3, and P + RR2 was 13.4%, 12.3%, and 12.0%, respectively, as compared to the control (Figure 6D).

### 2.9. Yield Parameters of Wheat Under Natural Conditions

Yield components of wheat, including shoot length, spike length, no. of spikes, and weight per 100 grains, were measured, and a clear decrease in these parameters was noticed under stressed conditions. However, treatment with compost and peat combined with PGPR significantly increased the yield parameters in both stressed and unstressed conditions. In the case of yield attributes, the maximum shoot length was observed by P + RR2, C + RR3, and P + RR3 in both stressed and unstressed conditions. The maximum spike length was observed in P + RR3 in an unstressed environment, while, under stress, C + RR2 yielded the maximum results. For the no. of spikes, P + RR2 gave the maximum results in unstressed conditions, while P + RR3 gave the maximum no. of spikes in stressed conditions. In the case of grain weight, P + RR3 gave the maximum results in both stressed and unstressed conditions (Table 8).

## 3. Discussion

Around 36% of the global population is dependent on wheat as a staple crop, and it is ranked the most grain-producing crop [18]. However, due to harsh climate fluctuations, the temperature of the Earth is rising 2–4 °C annually, resulting in different types of environmental stresses, including drought, salinity, and global warming [19]. The climate of Pakistan has been dynamically changing for the past few decades. This instability in the environment causes several abiotic stresses on crops. Salt stress is one of the major stresses that drastically affects crop productivity [20]. This study aimed to find a novel approach that can improve the production of wheat facing salt stress. After conducting a series of experiments, the findings revealed that bacterial application, in combination with carriers, could be a suitable approach for improving wheat growth under salt stress. The experiments conducted in this study showed that the use of plant growth-promoting rhizobacterial strains (*Bacillus wiedmannii* and *Bacillus paramobilis*) combined with peat and compost effectively helped plants cope with salt stress and increase the yield of wheat. These are the same findings as reported by other studies using other strains of *Bacillus*, including *Bacillus thuringiensis*, *B. subtilis*, *B. amyloliquefaciens*, and *B. velezensis* [21,22]. The application of *Bacillus* spp. against salinity to improve wheat growth has been investigated, but this study showed that the use of carriers like peat and compost makes these strains even more accessible and effective in both natural and field environments [23].

The most significant improvements were observed, on average, in the case of peat, rather than compost, in combination with RR3. However, RR2 gave better results with compost in most of the experiments. If comparisons were made between all the treatments and the control, then all the treatments gave better results as compared to the control. The halotolerant rhizobacterial strains have great potential to improve plant growth under a saline environment. This is consistent with other research showing that halotolerant bacteria have advantageous plant–microbe interactions and are well-suited to saline settings [24,25]. The improved growth and yield parameters and resistance toward salinity and drought by *Bacillus* sp. have been reported in the study [26]. As shown in this research, the *Bacillus* spp. have been reported to have many plant growth-promoting attributes such as the production of auxin, siderophores, and extracellular enzymes, phosphate solubilization, nitrogen fixation, metal resistance, and biocontrol activities [27,28,29,30].

These results are consistent with research showing that PGPR can improve plant development by producing phytohormones and solubilizing phosphate, which are essential for nutrient availability in environments. The strains (RR2) and (RR3) with plant growth-promoting attributes were selected for application on wheat to assess the efficiency of their plant–microbe interaction potential when combined with the selected carriers. These treatments improved the vegetative parameters, including root length, shoot length, fresh and dry root weight, and fresh and dry shoot weight, as compared to the control. Similar or greater effects produced by *Bacillus subtilis*, *Bacillus velenzensis*, and other *Bacillus* strains have been reported [31,32,33].

Moreover, salinity affects the chlorophyll content by damaging photosystem II and causing a decrease in the rate of photosynthesis in wheat through the closing of stomata due to imbalances of Na^+^ ions [34]. A recent study showed that different varieties of wheat are affected by salinity, resulting in the reduction of several photosynthetic parameters such as REL content, RWC content, rate of transpiration, and temperature. Salinity also affects the chlorophyll and carotenoid content of plants by the production of excessive reactive oxygen species and H_2_O_2_ [35]. However, the exogenous application of *Bacillus paramobilis* in combination with peat significantly increased the chlorophyll level and reduced the production of ROS. These results have been reported in several plants, including cucumber, rice, and tobacco [36,37].

The application of organic matter, such as compost, combined with PGPR helps in increasing the microbial activity in the soil and facilitates the breakdown and availability of organic matter to the microorganisms. The application of compost also enhances the water-holding capacity of soil, microbial respiration rate, and osmosis. It also balances the ionic concentration by increasing the uptake of K^+^ ions from the roots to the leaves, resulting in a decrease in the Na^+^ ion concentration by the production of auxin and exopolysaccharides [38,39]. A sandy loam composition with an alkaline pH of 8.6 and an electrical conductivity of 2.37 dS^−1^ was found by soil texture analysis. Wheat growth was greatly enhanced by the use of PGPR with both carriers as compared to the control. Another study highlighted the role of peat in balancing the ionic concentrations in soil by regulating the K^+^/Na^+^ ion ratio in the soil. The application of peat and another organic carrier like biochar increases K^+^ uptake in soil and also helps in the reduction of Na^+^ ions, ultimately coping with salt stress. More concentration of K^+^ ions facilitates plant growth and germination. The findings of the current research also follow the previous data, proving that the peat application increases the plant yield and helps plants cope with salinity stress, as compared to the control [40].

All of the above findings suggested that the application of PGPR in combination with peat and compost showed a significant improvement in the growth and yield of wheat as compared to the control in both unstressed and stressed conditions. This portable and novel approach could be used in the field for improved wheat crop production in salt-affected areas.

## 4. Materials and Methodology

### 4.1. Plant and Bacterial Strains

Seeds of *Triticum aestivum* (Akbar-19) were purchased from Punjab Seeds Corporation, located in the city of Khanewal, Pakistan. Bacterial strains were isolated and characterized at the Department of Microbiology and Molecular Genetics (MMG) at The Women University, Multan, Pakistan.

### 4.2. Sampling, Isolation, and Purification of Bacteria

Following the protocol, soil samples were collected from the rhizospheric soil of plants near the Indus basin of Pakistan under sterile conditions [41]. The initial sampling was done from the root zones of *Eriobotrya japonica*, *Citrus limon*, *Bauhinia variegate*, and *Syzygium cumini* growing in the district of Muzaffargarh (30.0736° N, 71.1805° E) and Multan (30.1864° N, 71.4886° E). The soil samples were carefully transferred into sterile zip lock bags and transported to the laboratory of the MMG department of The Women University, Multan. One gram of the soil sample was dissolved in 9 mL of saline and serially diluted. Dilutions (10^−3^ and 10^−5^) were spread on Luria–Bertani (LB) agar medium plates that were incubated at 37 °C for 24 h. Distinct colonies were purified using the quadrant streaking method.

### 4.3. Screening of Salt-Tolerant Strains

LB agar plates with salt concentrations (0–1500 mM) were used for the screening of salt-tolerant strains. Isolated colonies were picked based on minimum inhibitory conditions (MIC) of salt and re-streaked on LB agar with (850 mM) NaCl and incubated for 24 h at 37 °C [42]. Separated colonies were selected and stored at 4 °C for further study.

### 4.4. Identification of Salt-Tolerant Strains

Bacterial identification was done by performing basic biochemical tests according to Bergey’s manual system and 16S rRNA sequencing [43].

### 4.5. PGPR Characterizations

#### 4.5.1. Screening Based on Auxin Production

Salt-tolerant strains were screened for auxin production using Salowski’s reagent and following the protocol with some modifications [44]. The strains were incubated in LB broth with the addition of 0.1% L-tryptophan and incubated for 48 h in a shaker incubator at 120 rpm. The supernatant was extracted by using centrifugation at 10,000 rpm for 10 min. Then, 2 mL of supernatant was mixed with 2 mL of Salowski’s reagent and incubated for 30 min in the dark. The appearance of a pink to red color indicates the production of auxin. The quantification of auxin was done using a spectrophotometer. The optical densities (ODs) were noted at 530 nm. The standard graph method was used for the quantitative analysis using synthetic auxin solutions of different concentrations. The Gas Chromatography–Mass Spectrometry (GC–MS) analysis was done to validate indole compounds and their derivatives specifically [45].

#### 4.5.2. Screening Based on Phosphate Solubilization

The experiment involved assessing the phosphate-solubilizing ability of the isolated rhizobacteria using the Pikovskaya’s agar containing glucose, calcium phosphate (Ca_3_(PO_4_)_2_), ammonium sulfate ((NH_4_)_2_SO_4_), sodium chloride (NaCl), magnesium sulfate (MgSO_4_.7H_2_O), potassium chloride (KCL), yeast extract, manganese(II) sulfate monohydrate (MnSO_4_.H_2_O), ferrous sulfate heptahydrate (FeSO_4_.7H_2_O), agar, bromophenol blue, and distilled water. The bacteria were spot-inoculated on NBRIP agar plates and incubated at 30 °C. The solubilizing index was determined using a formula described in the study [46]. The quantification of solubilized inorganic phosphate was conducted using the standard curve method [47].

#### 4.5.3. Screening Based on Nitrogen Fixation

The ability of bacteria to fix nitrogen was determined by growing strains on Jensen’s media (N_2_-free media) containing sucrose, dipotassium phosphate, magnesium sulfate, NaCl, ferrous sulfate, sodium molybdate, calcium carbonate, and agar. Halo zones on the plates indicated the presence of N_2_-fixing bacteria. The N_2_-fixing capacity of the strains showed positive results determined using Nessler’s reagent and following the protocol [48].

#### 4.5.4. Screening Based on HCN, Ammonia, and Exopolysaccharide Production

A comprehensive screening of the salt-tolerant rhizobacteria was conducted to evaluate their capacity for hydrogen cyanide (HCN) production, ammonia production, and exopolysaccharide production, following the protocol mentioned earlier [49].

#### 4.5.5. Screening Based on the Production of Cell Wall-Degrading Enzymes

The production of chitinase, protease, and pectinase enzymes was evaluated by salt-tolerant rhizobacterial strains [50].

### 4.6. Characterization of Carrier Material

Peat and compost were selected for the experiment and purchased from Green Enterprises Pk. (GEP: Garden Gallery), located in Multan, Pakistan. The physicochemical analysis of peat and compost was also performed.

#### Stability of Bacteria in Carriers

A total of 10 mL of bacteria with 10^8^ colony forming units (CFU) per mL was thoroughly mixed with 50 g of autoclaved carrier and packed in ultraviolet [51]-sterilized polythene bags. These bags were prepared in and placed at room temperature (25–30 °C). CFU was evaluated at time intervals of 0 days, 1 month, 3 months, and 6 months.

$$\mathrm{Colony}\;\mathrm{forming}\;\mathrm{unit}\;=\frac{\mathrm{Number}\;\mathrm{of}\;\mathrm{colonies}\;\times \;\mathrm{Dilution}\;\mathrm{factor}}{\mathrm{Volume}\;\mathrm{of}\;\mathrm{inoculum}}$$

Using the above formula, CFU was evaluated after each interval to check the stability of the bacteria in the carriers [52].

### 4.7. Nutrient Analysis of Soil

Nutrient analysis of soil and carrier materials used for the experiments was done to evaluate nitrogen, potassium, and phosphorus concentrations, along with moisture content, electrical conductivity, and organic content, as described in [53].

### 4.8. Experimental Layout for Plant–Microbe Interaction

The efficiency of the selected strains in the presence of carriers was evaluated through their application to wheat under axenic and natural conditions (wire-house). Seeds of *Triticum aestivum* (variety Akbar-19) were purchased from Punjab Seed Corporation, Khanewal, Pakistan, sown on 1 November, and harvested in March. The experiment was performed at The Women University, Multan. Plant–microbe interaction was observed in the presence and absence of salinity stress, and 200 mM NaCl stress was applied [54].

#### Plant–Microbe Interaction

The initial experiment was performed to check the efficiency of the prepared treatment on wheat under controlled conditions in the laboratory of the MMG department. A total of 54 small cups (3.5″ × 2.6″) were filled 3/4 full with autoclaved soil and labeled accordingly (Table 9). The seeds were treated with 0.1% mercuric chloride for sterilization. After treatment, the seeds were coated with PGPR and carriers (peat and compost). Control seeds were dipped in autoclaved water for 30 min after sterilization. (T1 and T2) seeds, after sterilization, were dipped in broth for 30 min; the broth had a 24 h growth of the respective strains. (T3, T4, T5, T6, T7, T8) seeds were coated with respective carriers and PGPR + carrier combinations. Ten treated seeds were sown in each cup and placed in a completely randomized manner. A 12 h photoperiod with a (light intensity of 2200 lux) light chamber was used, and the cups were placed there for 15 days at 30 ± 2 °C temperature. After the germination of the seedlings, physiological parameters including shoot length, root length, fresh and dry shoot weight, and fresh and dry root weight were measured [55].

In the second phase, the experiment was performed under natural conditions (wire-house). For the wire-house experiment, 54 medium-sized pots were selected and filled with 11 kg of soil. The pots were labeled according to the treatment mentioned above, in triplicate, and placed in a Randomized Complete Block Design (RCBD). The experiment was conducted in two sets, one with no stress application and the other with 200 mM of salt stress. Seeds were sterilized, treated, and coated accordingly, as mentioned above, and sown in November in triplicate. Ten seeds were sown in each pot, and after germination, thinning was done from ten to five seeds per pot. Two weeks after germination, stress was applied. The physiological parameters (chlorophyll content, relative water content, relative electrolyte leakage, proline content, and total soluble sugar content) were evaluated. Yield parameters (shoot length, spike length, no. of spikes, no. of grains, and grain weight) were evaluated at the time of harvest.

### 4.9. Root Colonization Assay

To examine the root surface colonization by bacterial strains on wheat seedlings, scanning electron microscopy (SEM) analysis was performed [56]. The wheat seedlings were grown under controlled conditions in the laboratory using autoclaved soil. Treated wheat seedlings with PGPR were washed with double deionized water to remove any loosely adhered bacteria from the roots, and then the seedlings were fixed in 2.5% glutaraldehyde (*v*/*v*) for up to 4 h and dehydrated with a gradient of ethanol. The dried samples were coated with gold using an automated sputter coater for 3 min and carefully mounted for visualization. Treated samples were then examined under SEM using different magnifications.

### 4.10. Physiological Parameters

#### 4.10.1. Chlorophyll Content

To calculate the total chlorophyll and carotenoid content, 0.1 g of fresh leaves was mixed with 10 mL of 80% acetone for a night. After centrifugation at 10,000 rpm for 5 min, the optical densities of supernatants were noted at 663 nm, 645 nm, and 470 nm [57].

$$\mathrm{Chlorophyll}\;a=\frac{\left(12.7\times \mathrm{OD}663-2.69\times \mathrm{OD}645\right)\times 5}{1000\times 5}$$

$$\mathrm{Chlorophyll}\;b=\frac{\left(22.9\times \mathrm{OD}645-4.68\times \mathrm{OD}663\right)\times 5}{1000\times 5}$$

$$\mathrm{Carotenoids}=\frac{1000\times \mathrm{OD}470-3.27\times a-104\times b}{229}$$

#### 4.10.2. Relative Electrolyte Leakage (REL)

To determine REL, small parts of leaves from all treatments were cut into discs and dipped in double-distilled water for 4 h. Electrical conductivities were noted (EC1) using a digital device. The samples were then boiled in the water bath for 30 min and then cooled down. Electrical conductivities were noted (EC2), and REL was calculated using the following formula [58].

$$\%\;\mathrm{REL}\;=\frac{\mathrm{EC}1}{\mathrm{EC}2}\times 100$$

#### 4.10.3. Relative Water Content (RWC)

For the estimation of RWC, the weight of fresh leaves (FW) was measured immediately after sampling, and, after the saturation of leaves in water for 8 to 12 h at 4 °C, turgid weight (TW) was measured. Leaves were dried at 80 °C in a drying oven for 24 h, and dry weight (DW) was calculated using the following formula [59].

$$\mathrm{RWC}=\frac{\mathrm{FW}-\mathrm{DW}}{\mathrm{TW}-\mathrm{DW}}\times 100$$

### 4.11. Biochemical Parameters

For the estimation of proline content from the fresh leaves, the Bates method was used as described in [59]. The total soluble sugar content of leaves in wheat was calculated using the protocol mentioned in [60].

### 4.12. Yield Parameters

After harvesting the plants of the wire-house, yield parameters including length, spike length, number of spikes, number of grains, and weight of 100 grains per treatment were evaluated as mentioned in [61].

### 4.13. Statistical Analysis

Data obtained from the above-mentioned experiments was statistically evaluated through analysis of variance [62] using IBM SPSS statistics 25.0 software [62]. Tukey’s test with a significance level of *p* < 0.05 was used to check the presence of any significant difference between the treatments. All the statistical analyses were performed within the defined groups (stressed and unstressed) [63].

## 5. Conclusions

This research concludes that the combined application of the salt-tolerant strains *Bacillus wiedmannii* (RR2) and *Bacillus paramobilis* (RR3) with organic carriers has significantly enhanced wheat growth under saline conditions and has proven to be a sustainable approach for improving wheat yield. The pot experiments have demonstrated the effectiveness of these strains in promoting plant health in natural environments by increasing chlorophyll a and chlorophyll b content, proline and TSS content, and yield attributes of wheat, reinforcing their potential as viable biofertilizers. Additionally, these formulations have provided an economical and sustainable approach to improve bacterial stability, colonization efficiency, and long-term soil fertility. These findings have underscored the potential of integrating PGPR with organic amendments to develop cost-effective and eco-friendly strategies for enhancing crop resilience in salt-affected soils. Future research should focus on the molecular study of these strains to identify the genes that play a role in phytohormone production and salt tolerance. Moreover, in the future, field-scale validation of these treatments to facilitate their large-scale adoption in sustainable agriculture can be studied.

## Figures and Tables

**Table 1:** Sampling sites, soil texture, and plant source of rhizospheric samples.

Sr. No.	Sample Name	No. of Isolates	Plant Source	Soil Texture	Location
1.	MUL1	4	*Eriobotrya japonica*	Loamy soil	Multan
2.	MZG1	4	* Bauhinia variegate *	Sandy soil	Muzaffargarh
3.	MUL2	5	* Citrus limon *	Saline soil	Multan
4.	MZG2	6	* Bauhinia variegate *	Sandy soil	Muzaffargarh
5.	MUL3	4	* Citrus limon *	Saline soil	Multan
6.	MUL4	8	* Eriobotrya japonica *	Loamy soil	Multan
7.	MUL5	5	* Citrus limon *	Saline soil	Multan
8.	MZG3	3	* Bauhinia variegate *	Sandy soil	Muzaffargarh
9.	MZG4	5	* Syzygium cumini *	Loamy soil	Muzaffargarh
10.	MUL5	6	* Eriobotrya japonica *	Loamy soil	Multan

**Table 2:** Plant growth-promoting (PGP) attributes of salt-tolerant rhizobacterial strains.

Bacterial Strains	Auxin (µg/mL)	Phosphate (µg/mL)	Nitrogen Fixation Zones (cm)	HCN Production	Ammonia Production	EPS Production
RR1	41.0 ± 0.7	217.2 ± 0.3	1.13 ± 0.05	+	−	+
RR2	428.7 ± 0.5	1316.1 ± 0.8	1.33 ± 0.05	+++	++	++
RR3	351.1 ± 0.8	780.6 ± 0.5	2.13 ± 0.05	++	++	+
RR6	36.5 ± 0.5	200.1 ± 0.7	0.86 ± 0.05	−	−	−
RR8	455.7 ± 0.5	255.9 ± 0.3	2.43 ± 0.05	++	−	−

All values in the table are the mean of three replications ± SD. “+” indicates the presence of activity. “−” indicates the absence of activity. “+”, “++” and “+++” show intensity of the activity.

**Table 3:** Extracellular enzyme production by PGPR.

Salt-Tolerant Strains	Chitinase	Pectinase	Catalase	Protease	Amylase
RR1	+	−	−	−	−
RR2	+	−	−	+	+
RR3	+	+	−	+	−
RR6	+	−	−	−	+
RR8	−	−	−	+	+

“+” indicates the presence of activity. “−” indicates the absence of activity.

**Table 4:** IAA and its derivatives found in salt-tolerant PGPR.

Analytes	RR2	RR3
Indole acetic acid	−	+
Benzoic acid	−	+
n-Propyl acetate	−	+
Toluene	+	+
2-(Pyridin-2-ylformamido)acetic	+	−
4-(4-Hydroxy-2,5-dimethylbenzyl)	+	+
4-tert-Butylphenol	+	−
3-Trifluoroacetoxyhexadecane	−	+
4-Methyl-2-trimethylsilyloxy-acetone	+	−
Cyclopentasiloxane, decamethyl	−	+
Pyrrolo[1,2-a]pyrazine-1,4-dione	−	+
n-hexadeconic acid	−	+
Bis(2-ethylhexyl)s phthalate	+	+
Dichloroacetic acid, heptadecyl	−	+
Ticosene	−	+
Tris(tert-butyldimethylsilyloxy)	−	+
Arsenous acid, tris(rimethylsil)ester	+	+
Methyltris(trimethylsiloxy)silane	+	+
Tetrasiloxane, decamethyl	−	+
4-(7-Methyloctyl)phenol	+	+
Cyclotrisiloxane, hexamethyl	−	+
1,4-Bis(trimethylsilyl)benzene	−	+
Ethoxy(phenyl)silanediol	+	+

“+” indicates the presence of compound. “−” indicates the absence of a compound.

**Table 5:** Physiochemical analysis of the carrier’s compost and peat.

Parameters	Compost	Peat
Electrical Conductivity (dS^−1^)	2.15	1.93
pH	5.6	5.28
Nitrogen (%)	1.14	0.92
Phosphorus (%)	0.97	0.64
Potassium (PPM)	87.61	73.56
Moisture content (%)	3.89	3.73
Water-holding capacity (%)	28.42	31.12

**Table 6:** Bacterial population (1 × 10^7^ CFU mg^−1^) in compost and peat at 25 °C to 30 °C.

Carriers	Strains	Bacterial Population (1 × 10^7^ CFU mg^−1^) at Incubation Period (Days)						
		15	30	60	90	120	150	180
Compost	RR2-C	35.1 ± 0.1 ^c^	31.8 ± 0.6 ^d^	24.8 ± 0.2 ^c^	21.0 ± 0.7 ^d^	17.5 ± 0.6 ^d^	11.1 ± 0.9 ^b^	7.9 ± 1.1 ^b^
	RR3-C	30.4 ± 0.2 ^a^	27.8 ± 0.3 ^c^	213 ± 0.8 ^b^	18.2 ± 0.6 ^c^	14.8 ± 0.6 ^c^	10.8 ± 0.5 ^ab^	8.3 ± 0.7 ^b^
Peat	RR2-P	31.8 ± 0.7 ^b^	25.9 ± 0.7 ^b^	21.1 ± 0.9 ^b^	15.9 ± 0.1 ^b^	13.2 ± 0.1 ^b^	9.8 ± 0.3 ^ab^	7.4 ± 0.8 ^b^
	RR3-P	29.2 ± 0.6 ^a^	22.9 ± 0.6 ^a^	19.2 ± 0.2 ^a^	14.3 ± 0.2 ^a^	11.3 ± 0.2 ^a^	9.5 ± 0.2 ^a^	5.2 ± 0.5 ^a^

All data represented are the means of triplicate values. The ± values showed the standard error, and those that do not share a letter (a–d) are significantly different (*p* ≤ 0.05, Tukey’s test).

**Table 7:** Nutrient analysis of soil.

Parameters	Soil
EC (dS^−1^)	2.37
pH	8.6
Available nitrogen (mg kg)	91
Available phosphorus (mg kg)	4.42
Available potassium (mg kg)	98
Texture	Sandy loam
Organic matter (%)	0.25
Saturation (%)	11

**Table 8:** Yield attributes of wheat in the natural environment.

Treatments	Length (cm)	Spike Length (cm)	No. of Spikes	Weight per 100 Grains (g)	Length (cm)	Spike Length (cm)	No. of Spikes	Weight per 100 Grains (g)
	Unstressed				Stressed			
Control	55.0 ± 1.0 ^a^	8.1 ± 1.6 ^a^	13.3 ± 4.0 ^a^	3.20 ± 0.1 ^a^	44.3 ± 3.7 ^a^	8.0 ± 1.7 ^a^	9.0 ± 2.6 ^a^	2.90 ± 0.1 ^a^
T1	65.6 ± 1.5 ^c^	10.3 ± 1.5 ^c^	16.0 ± 3.0 ^b^	4.50 ± 0.01 ^b^	64.6 ± 4.1 ^c^	9.3 ± 0.5 ^a^	12.3 ± 2.0 ^b^	3.96 ± 0.1 ^de^
T2	63.3 ± 0.5 ^b^	10.0 ± 1.7 ^c^	16.3 ± 2.0 ^b^	3.83 ± 0.05 ^a^	64.3 ± 4.0 ^c^	9.6 ± 0.5 ^a^	16.6 ± 0.5 ^c^	3.20 ± 0.1 ^b^
T3	62.6 ± 5.5 ^b^	10.6 ± 0.5 ^c^	13.3 ± 2.3 ^a^	4.60 ± 0.01 ^b^	63.3 ± 1.5 ^c^	10.3 ± 0.5 ^b^	13.3 ± 3.7 ^b^	3.53 ± 0.05 ^c^
T4	67.0 ± 2.6 ^d^	10.9 ± 1.7 ^c^	17.0 ± 1.0 ^c^	4.82 ± 0.02 ^b^	68.6 ± 4.5 ^c^	10.0 ± 1.7 ^b^	16.6 ± 3.7 ^c^	3.70 ± 0.1 ^c^
T5	68.3 ± 1.5 ^e^	11.0 ± 0.00 ^c^	16.0 ± 1.0 ^b^	4.94 ± 0.06 ^b^	67.3 ± 4.1 ^c^	9.6 ± 1.1 ^a^	19.6 ± 4.6 ^d^	4.03 ± 0.05 ^ef^
T6	62.0 ± 2.0 ^b^	9.0 ± 1.7 ^b^	14.3 ± 3.2 ^a^	5.13 ± 0.05 ^c^	57.6 ± 4.5 ^b^	9.6 ± 1.1 ^a^	17.0 ± 6.9 ^cd^	4.23 ± 0.05 ^fg^
T7	68.0 ± 2.0 ^e^	11.0 ± 1.7 ^c^	21.0 ± 7.0 ^d^	5.23 ± 0.05 ^c^	65.6 ± 3.5 ^c^	10.3 ± 0.5 ^b^	19.0 ± 1.0 ^d^	4.46 ± 0.05 ^g^
T8	67.3 ± 2.0 ^d^	12.0 ± 1.0 ^d^	17.6 ± 4.1 ^c^	5.86 ± 0.05 ^c^	68.6 ± 4.5 ^c^	10.0 ± 1.0 ^b^	28.0 ± 6.0 ^e^	4.76 ± 0.05 ^h^

All data represented are the means of triplicate values. The ± values showed the standard error, and those that do not share a letter (a–h) are significantly different (*p* ≤ 0.05, Tukey’s test).

**Table 9:** The following are the treatments for the experiment.

Name	Unstressed	Stressed (200 mM)
Control	No PGPR + No carrier	No PGPR + No carrier
T1	PGPR (RR-2)	PGPR (RR-2)
T2	PGPR (RR-3)	PGPR (RR-3)
T3	Compost (C)	Compost (C)
T4	C + RR-2	C + RR-2
T5	C + RR-3	C + RR-3
T6	Peat (P)	Peat (P)
T7	P + RR-2	P + RR-2
T8	P + RR-3	P + RR-3

## Data Availability

The original contributions related to the study are included in the article; further inquiries can be directed to the corresponding author.
